# Hypovirulence caused by mycovirus in *Colletotrichum fructicola*

**DOI:** 10.3389/fpls.2022.1038781

**Published:** 2022-10-07

**Authors:** Jun Guo, Xinyu Zhou, Fangling Xie, Junjie Cao, Shuangqing Liu, Jie Zhong, Hongjian Zhu

**Affiliations:** Hunan Provincial Key Laboratory for Biology and Control of Plant Diseases and Insect Pests, Hunan Agricultural University, Changsha, China

**Keywords:** *Colletotrichum fructicola*, CgOLV1-CfOLV1, CfOLV2, the integrity of the cell wall, transcriptomic analysis

## Abstract

*Colletotrichum fructicola* is a pathogenic fungus causing leaf black spot and fruit rot disease in a wide variety of crops. Some mycoviruses that cause detrimental effects on fungal hosts could be useful in studying the pathogenesis of fungal hosts. In this study, we reported two mycoviruses, *Colletotrichum fructicola* ourmia-like virus 1- *Colletotrichum gloeosporioides* ourmia-like virus 1 (CfOLV1-CgOLV1) and *Colletotrichum fructicola* ourmia-like virus 2 (CfOLV2), from a *C. fructicola* fungus. The complete genome sequences of CfOLV1-CgOLV1 and CfOLV2 contain 2,516 bp and 2,048 bp, respectively. Both of these viruses contain only one open reading frame (ORF), which encodes an RNA-dependent RNA polymerase (RdRp). CfOLV1-CgOLV1 was identical as the previously reported virus CgOLV1. Phylogenetic analysis showed that CfOLV2 is closely related to *Scleroulivirus* and *Magoulivirus* in the family Botourmiaviridae. Virus elimination and horizontal transmission experiments proved that the associated mycoviruses could reduce the pathogenicity of the host *C. fructicola*. In addition, we found that the virus-containing strains showed a much higher percentage of appressorium formation and more melanin production compared to isogenic virus-free strain, and the presence of the virus is detrimental to the growth of host fungi and regulates the integrity of the cell wall. Transcriptomic analysis showed that mycovirus infection caused various abnormal genes expression in *C. fructicola*. To the best of our knowledge, this is the first report of a hypovirulence-associated ourmia-like mycovirus in *C. fructicola*.

## Introduction

Mycoviruses (fungal viruses) are viruses that infect and replicate in almost all fungal groups (Ghabrial and Suzuki, 2009; Pearson et al., 2009). Since the first report of mycoviruses infecting *Agaricus bisporus* in 1962, an increasing number of mycoviruses have been reported in recent years (Hollings, 1962). Generally, most mycoviruses are cryptic in their entire growth history and show symptomless effects on their host fungi. However, some mycoviruses can reduce or enhance the virulence of host fungi (Nuss, 2005; Pearson et al., 2009; Yang et al., 2018). Therefore, these viruses are given the name of hypovirulence-associated viruses, such as *Cryphonectria hypovirus* 1 (CHV1), *Sclerotinia sclerotiorum* hypovirulence-associated DNA virus 1 (SsHADV1), *Botrytis cinerea* hypovirus 1 (BcHV1) and *Alternaria alternata* hypovirus 1 (AaHV1), while the viruses enhancing host virulence were called hypervirulence viruses, such as *Talaromyces marneffei* partitivirus-1 (TmPV1) (Hollings, 1962; Yu et al., 2010; Hao et al., 2018; Lau et al., 2018; Li et al., 2019a). Accordingly, CHV1 was reported to be successfully used to control chestnut blight in Europe, and SsHADV1 is effective in controlling rape sclertiniose in the field. Therefore, mycoviruses are considered potential biological control agents for crop fungal diseases (Anagnostakis et al., 1986; Heiniger and Rigling, 1994; Chen and Nuss, 1999; Zhang et al., 2020).

In general, there are three genomic types of mycoviruses: double-stranded RNA (dsRNA), single-stranded RNA (ssRNA), and single-stranded DNA (ssDNA) (Yu et al., 2010; Du et al., 2014; Liu et al., 2014; Ghabrial et al., 2015). The ssRNA mycoviruses were divided into several families, including Alphaflexiviridae, Deltaflexiviridae, Gammaflexiviridae, Tymovirales, Barnaviridae, Botourmiaviridae, Endornaviridae, Hypoviridae, Mitoviridae, Narnaviridae, Metaviridae and Pseudoviridae (Kotta-Loizou, 2021; Nerva and Chitarra, 2021). The RNA genome of viruses in Narnaviridae is extremely simple in that it contains only a single RNA segment that is 2.3-3.6 Kb in length, encoding only RNA-dependent RNA polymerase (RdRp) for its own replication (Hillman and Cai, 2013). Thus, there is no capsid to package nucleic acids in Narnaviridae viruses. Strikingly, even though *Narnavirus* and *Mitovirus* belong to Narnaviridae, the virus replication sites are different. Several lines of evidence indicate that *Narnavirus* replicates in the host cytoplasm, *Mitovirus* replicates in host mitochondria, and *Mitovirus* often applies mitochondrial genetic codes (Polashock and Hillman, 1994). For example, the “UGA” triplet is regarded as a stop codon when applied to the nucleus, but in the fungal mitochondrial gene coding system, the “UGA” triplet translates into tryptophan (Mizutani et al., 2018). Phylogenetically, viruses in Narnaviridae contain three RNA fragments encoding RdRp, coat protein (CP) and movement protein (MP), which are most closely related to plant *Ourmiaviruses* (Brown, 1997; Rastgou et al., 2009; Hull, 2014). Interestingly, recent studies found that a class of mycoviruses (ourmia-like viruses) exhibit the closest phylogenetic relationship with plant *Ourmiaviruses* rather than *Narnavirus* or *Mitovirus* (Wang et al., 2020; Zhou et al., 2020). Thus, the existing classification system is unable to correctly classify this virus. Then, the International Committee on Taxonomy of Virus (ICTV) created a new family Botourmiaviridae comprising four genera (*Botoulivirus*, *Ourmiavirus*, *Scleroulivirus*, and *Magoulivirus*) to cover ourmia-like viruses (Ayllón et al., 2020). The genome of ourmia-like viruses contains one large segment encoding RdRp for its replication, and whether another segment exists that is similar to *ourmiaviruses* remains unknown.

*Colletotrichum* is a class of pathogenic fungi that can cause great economic loss and infect almost all field crops (Cannon et al., 2007). *Colletotrichum fructicola* was first reported on coffee berries in Thailand and has gradually been found on pear and other plants in recent years (Prihastuti et al., 2009; Weir et al., 2012; Huang et al., 2013; Li et al., 2013). Currently, several mycoviruses have been reported from *Colletotrichum*, such as *Colletotrichum acutatum* partitivirus 1 (CaPV1) (Zhong et al., 2014), *Colletotrichum higginsianum* Non-segmented dsRNA virus 1 (ChNRV1) (Campo et al., 2016), *Colletotrichum truncatum* partitivirus 1 (CtParV1) (Marzano et al., 2016), *Colletotrichum gloeosprioides* chrysovirus 1 (CgCV1) (Zhong et al., 2016), *Colletotrichum camelliae* filamentous virus 1 (CcFV-1) (Jia et al., 2017), *Colletotrichum fructicola* chrysovirus 1 (CfCV1) (Zhai et al., 2018), and *Colletotrichum gloeosporioides* ourmia-like virus 1 (CgOLV1) (Guo et al., 2019). Therefore, *Colletotrichum* might host a variety of mycoviruses.

Here, we identified and characterized two mycoviruses, named *Colletotrichum fructicola* ourmia-like virus 1- *Colletotrichum gloeosporioides* ourmia-like virus 1 (CfOLV1-CgOLV1) and *Colletotrichum fructicola* ourmia-like virus 2 (CfOLV2). CfOLV1-CgOLV1 is 100% identical to the previous reported virus and CfOLV2 is the new member in the family Botourmiaviridae. Meanwhile, we found that the presence of CfOLV2 together with dsRNA-M can cause hypovirulence in host fungi. The virus is detrimental to the growth of the host fungus and is able to regulate the integrity of the cell wall. Further, we also found virus-infected strain show a much higher percentage of appressorium. In order to elucidate the biological significance, we performed transcriptome analysis of virus-infected strain and congenic virus-cured strain.

## Materials and methods

### Fungal strain and cultural conditions

Strain CSG2-3 of *C. fructicola* was collected from infected citrus in Changsha, Hunan Province, China. The strain was purified by single conidia isolation. All the strains, including those with virus infection and virus-free strains, were maintained at 25°C in the dark on potato dextrose agar (PDA) medium.

### Extraction and purification of dsRNAs

Viral dsRNA was extracted from mycelia using the cellulose (CF-11) chromatography method, as described by Morris and Dodds (Morris and Dodds, 1979). To prepare the mycelium of CSG2-3, mycelium agar plugs were inoculated in potato dextrose broth (PDB) at 27°C with orbital shaking at 180 rpm for 5 days, and then the mycelium was harvested and stored at 80°C before use. The extracts were digested with RNase-free DNase I and S1 nuclease (Takara, Dalian, China) for elimination of DNA and single-stranded RNAs. The purified dsRNA was detected by agarose gel electrophoresis (1.0%, wv) and visualized by an AlghaImager HP gel imaging system (ProteinSimple, Silicon Valley, CA, USA) after staining with 0.1 mg/ml ethidium bromide.

### cDNA cloning and sequencing

Purified dsRNA was used as a template for cDNA cloning. The cDNA library was constructed using the methods described by Zhong (Zhong et al., 2016). Random primers (5’-GCCGGAGCTCTGCAGAATTCNNNNNN-3’) and specific primers (5’-GCCGGAGCTCTGCAGAATTC-3’) were used for reverse transcription and PCR amplification. The intermediate sequences were amplified with specific primers designed according to the obtained sequences, and the 5’ and 3’ terminal sequences were obtained using adaptor-ligated methods as previously reported (Xie et al., 2011; Zhong et al., 2016). All amplicons were cloned into the pMD18-T vector (Takara, Dalian, China) and transformed into *Escherichia coli DH5α* (TransGen Biotech, Beijing, China). Positive clones were selected for Sanger sequencing, with each base pair sequenced at least three times.

### Sequence analysis

Sequences were assembled using DNAMAN software version 6.0. The open reading frames (ORFs) in the full-length viral sequence were identified using the ORF Finder program with standard codon usage (https://www.ncbi.nlm.nih.gov/orffinder/). The Blastn and Blastp programs were used for homologous searches in the National Center for Biotechnology Information (NCBI) database (https://www.ncbi.nlm.nih.gov/). Clustal X was used for sequence alignment (Thompson et al., 1997). A phylogenetic tree was constructed using MEGA software version 6.0 (Tamura et al., 2013), and 1000 bootstraps analysis was used to determine the confidence value for each branch using neighbor-joining methods (Felsenstein, 1985; Saitou and Nei, 1987). The predicted secondary structures of 5’ and 3’ was measured by the Mfold program.

### Virus elimination and horizontal transmission of dsRNA

To investigate the biological effect of the virus and obtain a virus-free strain in *C. fructicola* strain CSG2-3, the protoplast regeneration technique was conducted as previously reported (Zhong et al., 2014; Kamaruzzaman et al., 2019) with minor modifications, mycelium agar plugs were inoculated in 250 mL PDB with orbital shaking at 180 rpm at 27°C for 3-5 days. Then, fresh mycelium was collected and placed in the enzymatic hydrolysate (1% Lysing Enzymes, 0.01% Snailase, 0.01% Driselase). The enzymatic hydrolysate was gently shaken (80-110 rpm) at 30°C and protoplast release was observed every two hours. When the protoplast was released well, the enzymatic hydrolysate was passed through three layers of mirror wiping paper, and the filtrate was collected. The filtrate was centrifuged at 4°C at 8000×g for 8 min, washed twice with 0.7 M MgSO_4_, and suspended in 200 µL STC buffer (1 mol/L sorbitol, 100 mM/L Tris-HCl pH=8.0, 100 mM/L CaCl_2_). Finally, the suspension was diluted and plated on yeast extract-bactotryptone-saccharose (YEPS) medium at 25°C for 3 days, and a single colony was transferred into PDA medium for colony morphology observation, dsRNA extraction and RT-PCR detection.

For virus horizontal transmission, we chose CSG2-3 as the donor and CSG2-3-Y7 as the recipient. Each mycelial agar plugs from CSG2-3 and CSG2-3-Y7 was placed approximately 1 cm apart on PDA media. After 7 days of pairing culture, the hyphal agar plugs close to CSG2-3-Y7 and away from CSG2-3-Y7 were transferred to PDA media. After three subcultures, the transformants were used for colony morphology observation, dsRNA extraction and RT-PCR detection.

### Growth rate and pathogenicity determination

The colony morphology and growth rate of the virus-infected and virus-free strains were assessed. Fresh mycelium agar plugs were taken under the same conditions and transferred to PDA media at 27°C in the dark. Each strain was tested three times. After 7 days, each colony diameter was measured and assessed.

For the pathogenicity test, apples were used to test the pathogenicity of virus-infected and virus-free strains. Before inoculation, the growth conditions of all strains should be consistent, and the surface of the apple should be disinfected with 70% alcohol. Several wounds were made on the surface of the apple by a sterile needle, and then the same size (approximately 0.8 cm) of mycelial agar plugs and 10 μL conidial suspension (6×10^5^ conidia/mL) were inoculated on the wound surface of the apple. After inoculation, all inoculated apples were placed into a contaning box with high temperature (26°C) and humidity (>85%), with three biological replicates each. Seven to ten days later, the severity was observed, and each inoculated apple lesion diameter was measured.

Data on growth rate and pathogenicity were collected and analyzed by using SPSS software (IBM SPSS statistics 20). Means of mycelial growth rate and lesion diameter on apple and CSG2-3, CSG2-3-Y7, CSG2-3-F8, CSG2-3-F10 and CfOLV1-transfected derivatives (CSG2-3-Y7-D3, CSG2-3-Y7-D4) were compared using one-way analysis of variance (ANOVA). Treatment means of mycelial growth rates on PDA in the colony morphology trials on five different media, including conidial production, lesion diameters, and appressorium formation, were compared using Student’s *t*-test at the α=0.05 level.

### Appressorium formation assays

An appressorium formation assay was performed as described by (Chen et al., 2020; Li et al., 2020) with few modifications. The conidial suspension was collected by filtration with three layers of mirror wiping paper, centrifuged at 10000 rpm for 3 min and washed twice with sterile water. The concentration of conidial suspension was adjusted to 6 x 10^5^ conidia/mL, and 100 μL conidial suspension was taken and incubated at 26°C in the dark on concave slides. The percentages of conidia-forming appressoria were measured by microscopic examination of at least 200 conidia. Each experimental replicate was performed three independent times.

### Total RNA extraction, transcriptomic analysis and real-time quantitative PCR

Mycelium agar plugs of CSG2-3 and CSG2-3-F10 were inoculated in 100 mL potato dextrose broth (PDB) with orbital shaking at 180 rpm at 27°C for 5 days. Then, total RNA was extracted from each strain using TRIzol according to the manufacturer’s protocol (TransGen, Changsha, China), and three biological replicates per strain were prepared. The samples were sent to Genedenovo Biotechnology Co., Ltd. (Guangzhou, China) for cDNA library construction and transcriptomic data analysis. Specifically, total RNA was delivered to the company and enriched by Oligo(dT) beads. The enriched mRNA fragments were then reduced to short fragments by fragment buffer and reversed into cDNA using random primers. The cDNA fragment was purified using the QiaQuick PCR extraction kit (Qiagen, Venlo, The Netherlands), the end was repaired, poly(A) was added, and the Illumina sequencing adaptor was attached. The ligation products were amplified by PCR and sequenced using Illumina HiSeq 2500 by Gene Denovo Biotechnology Co. (Guangzhou, China). High-quality reads were mapped to the reference genome (ncbi_GCF_000319635.1), and mRNA differential expression analysis was performed by DESeq2 (Love et al., 2014) software between two different groups (and by edgeR (Robinson et al., 2010) between two samples). Genes with a false discovery rate (FDR) below 0.05 and absolute fold change≥2 were considered differentially expressed genes/transcripts.

To verify the reliability of the transcriptome data, total RNA was extracted from CSG2-3 and CSG2-3-F10, and RT-qPCR was conducted with gene-specific primers. Reverse transcription was performed using a Maxima H Minus First Strand cDNA Synthesis Kit with a dsDNase kit (Thermo, Shanghai, China). RT-qPCR was performed using a SYBR Green Premix *Pro taq* HS qPCR kit (AG, Changsha, China) according to the manufacturer’s protocol, and the conditions were as follows: 30 s at 95°C, followed by 40 cycles at 95°C for 5s and 60°C for 30 s. The relative gene expression was analyzed by a CFX96™ manager system (Bio-Rad, Hercules, CA, USA) in 20 µL reactions. Gene expression was calculated using the 2^−ΔΔCt^ method and the actin gene as an internal reference gene. Each treatment contained three independent biological and technical replicates.

## Results

### Viral dsRNA in *C. fructicola*

**Figure 1:**
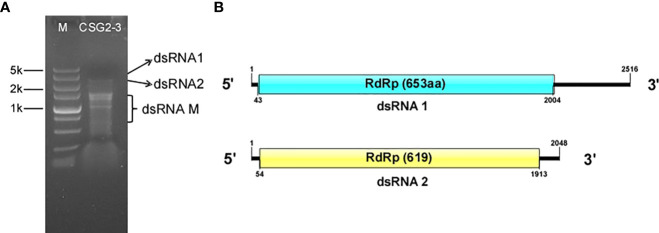
Agarose gel electrophoresis analysis and putative genome structure of dsRNA in *C*. *fructicola* strain CSG2-3. **(A)** 1% agarose electrophoresis analysis of dsRNA extracted from the mycelium of *C. fructicola* strain CSG2-3. M: Marker. CSG2-3: dsRNA extracted from CSG2-3. **(B)** Genome structure of dsRNA1 (up) and dsRNA2 (down).

To identify the mycoviruses in C. fructicola, viral dsRNA was extracted from the mycelium of C. fructicola strain CSG2-3 collected from infected citrus using the cellulose (CF-11) chromatography method, and then the dsRNA was subjected to 1% agarose gel electrophoresis. Several bands, ranging from 1.0 to 2.5 kbp, were observed under UV light (**Figure 1A**) and were temporarily designated dsRNA1, dsRNA2, and dsRNA-M.

### Nucleotide Sequence of the dsRNA in *C. fructicola*

The complete fragments of dsRNA1 and dsRNA2 were obtained by cDNA cloning. According to DNA sequencing, full-length dsRNA1 is 2,516 base pairs (bp) in length, with 49% GC content. Sequence basic local alignment search tool (BLAST) showed that dsRNA1 is 100% identical to the mycovirus CgOLV1, which indicates that dsRNA 1 is considered a species of CgOLV1, designated CfOLV1-CgOLV1. Notably, CSG2-3 differs from the host strain T2 of CgOLV1 in that the former is from citrus and the latter is from cotton.

The full-length cDNA sequence of dsRNA2 is 2,048 bp, with 51% guanine-cytosine (GC) content, and it contains a large open reading frame (ORF) putatively encoding a 619-aa protein with a molecular mass of approximately 71.6 kDa (**Figure 1B**). In addition, the 5’ and 3’ untranslated regions (UTRs) of dsRNA2 were 53 and 135 bp long, respectively. Subsequent analysis indicated that dsRNA2 is a new ourmia-like virus, which is designated *Colletotrichum fructicola* ourmia-like virus 2 (CfOLV2). The genome sequences of CfOLV1-CgOLV1 and CfOLV2 were submitted to the GenBank database with accession numbers MW300427 and MW300428, respectively. However, since no sequence information related to dsRNA-M has been detected, dsRNA-M is not discussed in this manuscript.

### Genome structure and phylogenetic analysis of CfOLV2

**Figure 2:**
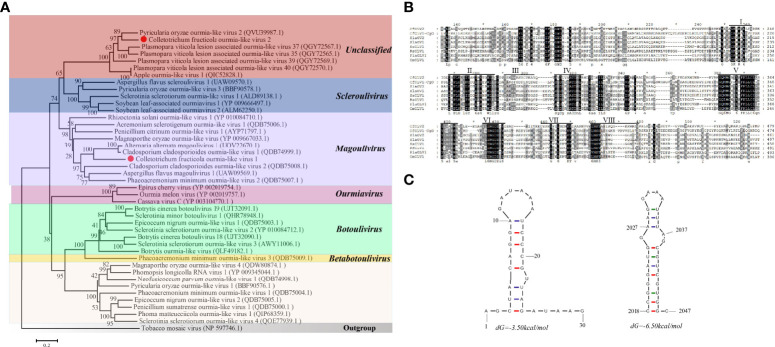
Phylogenetic tree, conserved motifs, and secondary structure prediction of CfOLV2. **(A)** A phylogenetic tree was constructed based on the amino acid sequences of RdRp using the neighbor-joining method in MEGA 6.0. Tobacco mosaic virus was used as an outgroup. The scale bar represents a genetic distance of 0.2 amino acid substitutions per site, and the red dots indicate the positions of CfOLV1 and CfOLV2. **(B)** Amino acid sequence alignment of the RdRp motifs and other selected ourmia-like viruses. The conserved motifs are indicated by Roman numbers. **(C)** Stem−loop structures could be predicted in the 5’ and 3’ UTRs of CfOLV2 with ΔG values of -3.50 and -6.50 kcal/mol, respectively.

A homology search in BLASTp showed that CfOLV2 has a significant relationship with other ourmia-like viruses. To investigate the taxonomic status of CfOLV2, a phylogenetic tree using the neighbor-joining method was constructed based on the RdRp sequence of CfOLV2 and other mycoviruses, such as *Mitovirus*, *Narnavirus*, *Ourmiavirus* and ourmia-like virus. The results showed that CfOLV2 is closely related to *Scleroulivirus* and *Magoulivirus* in the family Botourmiaviridae. However, CfOLV2 was classified into the same cluster as *Pyricularia oryzae* ourmia-like virus 2, *Plasmopara viticola* lesion-associated ourmia-like virus 35, 37, 39, 40 and Apple ourmia-like virus 1, which formed an independent branch (**Figure 2A**). Furthermore, a Conserved Domain Database (CDD) search and multiple protein alignment confirmed that the CfOLV2 protein has eight motifs that are characteristic of (+) ssRNA viruses RdRps (**Figure 2B**) (Koonin, 1991). In addition, the secondary structure of CfOLV2 was predicted with the Mfold program, and the results showed that CfOLV2 folds into a stable stem−loop structure with 5’ UTR and 3’ UTR ΔG values of 3.50 kcal/mol and 6.50 kcal/mol, respectively (**Figure 2C**).

### Biological analysis

**Figure 3:**
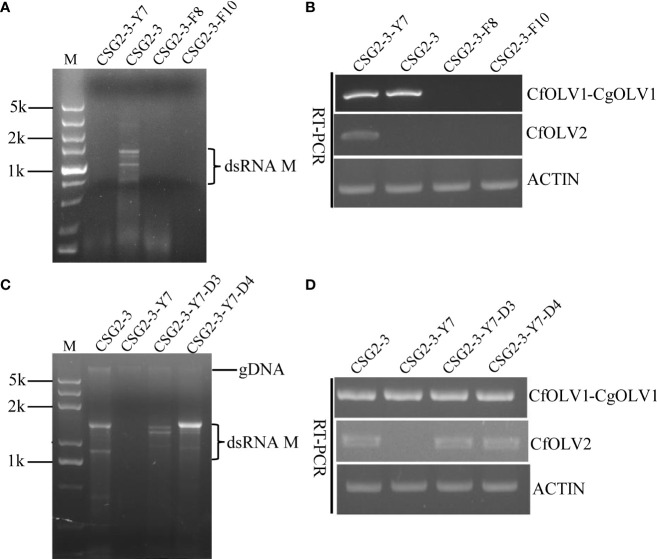
Agarose gel electrophoresis of dsRNA in *C. fructicola* and RT-PCR detection of CfOLV1-CgOLV1 and CfOLV2. **(A, C)** 1% agarose electrophoresis analysis of dsRNA extracted from the mycelia of CSG2-3, CSG2-3-Y7, CSG2-3-F8/F10, and CSG2-3-Y7-D3/D4. M=DL5000 DNA marker (TSINGKE, Changsha, China). **(B, D)** RT-PCR detection of CfOLV1-CgOLV1 and CfOLV2 in different isogenic strains.

To further understand the effects of the abovementioned virus on the host fungus *C. fructicola*, isogenic virus-free strains were obtained through protoplast regeneration and named CSG2-3-Y7, CSG2-3-F8 and CSG2-3-F10. When RNA segments were detected in these strains after dsRNA extraction, no RNA segment was found in CSG2-3-Y7, CSG2-3-F8 or CSG2-3-F10 (**Figure 3A**). However, real-time polymerase chain reaction (RT-PCR) showed that CSG2-3-Y7 contained only CfOLV1-CgOLV1, while there were no CfOLV1-CgOLV1 in CSG2-3-F8 and CSG2-3-F10 (**Figure 3B**). In addition, isogenic strains were also obtained by horizontal transmission experiments in which CSG2-3 was used as the donor strain and CSG2-3-Y7 as the recipient strain and named CSG2-3-Y7-D3 and CSG2-3-Y7-D4. Similarly, dsRNA extraction and RT-PCR verification were also performed. Surprisingly, RT-PCR results showed that CfOLV2 was successfully transferred from CSG2-3 into CSG2-3-Y7-D3 and CSG2-3-Y7-D4; moreover, electrophoresis results showed that dsRNA-M was also transferred into CSG2-3-Y7-D3 and CSG2-3-Y7-D4 (**Figures 3C, D** **)**.

**Figure 4:**
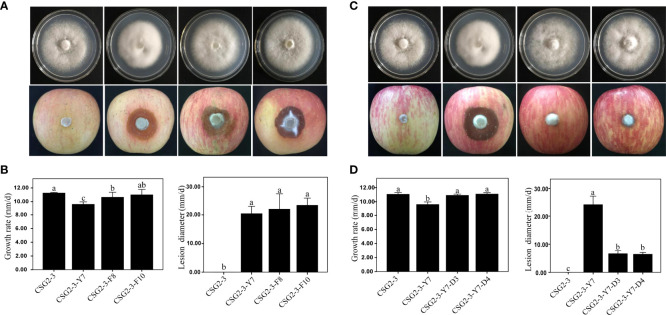
Growth rate and pathogenicity of the isogenic *C. fructicola* strain. **(A, C)** Colony morphology (up) and pathogenic situation on apples (down) of CSG2-3, CSG2-3-Y7, CSG2-3-F8/F10, and CSG2-3-Y7-D3/D4. **(B, D)** Growth rate (left) and pathogenicity (right) of CSG2-3, CSG2-3-Y7, CSG2-3-F8/F10 and CSG2-3-Y7-D3/D4. The different letters on the bars indicate significant differences at the α=0.05 level of confidence according to one-way analysis of variance (ANOVA).

The growth rate and pathogenicity of the virus-infected *C. fructicola* strain CSG2-3 were significantly different from the growth rate and pathogenicity of CSG2-3-Y7 and CSG2-3-F10. As shown in **Figure 4**, in terms of growth rate, CSG2-3-Y7 and CSG2-3-F8 were much lower than CSG2-3, while there was no significant difference between CSG2-3-F10 and CSG2-3, indicating that the virus has a certain influence on the growth rate of *C. fructicola* (**Figures 4A, B** **)**. The lesion diameters of apples infected by CSG2-3-Y7, CSG2-3-F8 and CSG2-3-F10 were much larger than CSG2-3, and there was no significant difference among CSG2-3-Y7, CSG2-3-F8 and CSG2-3-F10 (**Figures 4A, B** **)**. Therefore, the pathogenicity test preliminarily proved that the hypovirulence of CSG2-3 results from infection with CfOLV2 or dsRNA-M. We also compared the growth rate and pathogenicity among CSG2-3, CSG2-3-Y7, CSG2-3-Y7-D3 and CSG2-3-Y7-D4. Although CSG2-3, CSG2-3-Y7-D3 and CSG2-3-Y7-D4 showed the same characteristics in colony morphology and growth rate, strikingly, these strains showed significant differences in pathogenicity: the lesion diameters of apples infected by CSG2-3-Y7-D3 and CSG2-3-Y7-D4 were much smaller than CSG2-3-Y7 but larger than CSG2-3 (**Figures 4C, D** **)**. Overall, these data proved that the hypovirulence of CSG2-3 was associated with infection with CfOLV2 or dsRNA-M.

### Viral infections modulate appressorium formation and the conidia production of *C. fructicola*

**Figure 5:**
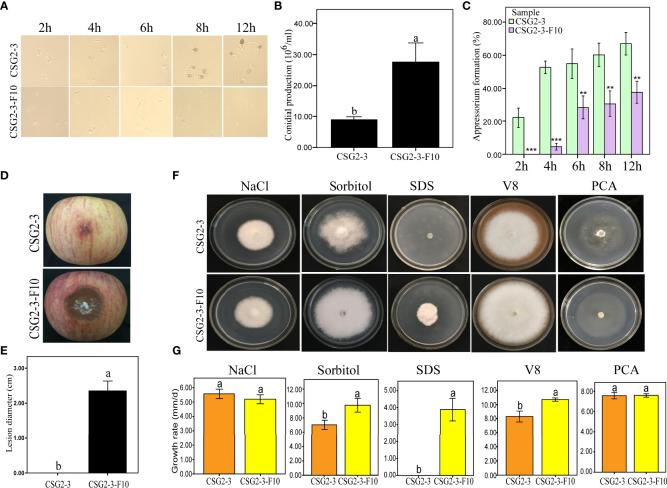
Growth and development of *C*. *fructicola* affected by virus infection. **(A)** Conidia were incubated on concave slides, and the samples were observed at different time points. **(B)** Conidial production of CSG2-3 and CSG2-3-F10. The different letters on the bars indicate significant differences at the α=0.05 level of confidence according to Student’s *t*-test. **(C)** The appressorium formation rates of CSG2-3 and CSG2-3-F10. The asterisks represent significant differences of CSG2-3 and CSG2-3-F10 at different periods (Student’s *t*-test, ‘***’, P<0.001 ‘**’, P<0.01). **(D)** The pathogenicity symptoms of CSG2-3 and CSG2-3-F10 conidial suspensions (6×10^5^ conidial/mL). **(E)** The pathogenicity of CSG2-3 and CSG2-3-F10 conidial suspensions. The different letters on the bars indicate significant differences at the α=0.05 level of confidence according to Student’s *t*-test. **(F)** Phenotypic growth of CSG2-3 and CSG2-3-F10 on V8 media, PCA media, and potato dextrose agar (PDA) media containing 1 M NaCl, 1 M sorbitol, and 0.1% SDS components. Colonies were grown on PDA for 7 days and photographed. **(G)** Growth rates of CSG2-3 and CSG2-3-F10 on different media. Different letters indicate a significant difference at the α=0.05 level of confidence according to Student’s *t*-test.

Both conidia and appressorium play an important role in the pathogenesis of *Colletotrichum* (Perfect et al., 1999; O'Connell et al., 2012), so it is important to observe whether viral infection affects conidia and appressorium formation in *Colletotrichum*. We found that the virus-free *C. fructicola* strain CSG2-3-F10 showed delayed appressorium formation and higher conidia production than the virus-infected strain CSG2-3 (**Figures 5A, B**). Moreover, the percentage of appressorium formation was much lower in the virus-free strain CSG2-3-F10 than in the virus-infected strain CSG2-3 at 2 hpi, 4 hpi, 6 hpi, 8 hpi and 12 hpi (**Figure 5C**). Interestingly, melanin was obviously observed at 6 hpi in CSG2-3 and gradually deepened at 8 hpi and 12 hpi, while melanin was not found in CSG2-3-F10 (**Figure 5A**). Notably, when we inoculated apples with conidial suspensions of the above strains, the pathogenicity of CSG2-3 was lower than that of CSG2-3-F10 (**Figures 5D, E**). Finally, we investigated whether the virus could affect fungal growth under different stress conditions. Five different media were made, including 1 M NaCl, 1 M sorbitol, 0.1% sodium dodecyl sulfate (SDS), V8 juice agar and plate count agar (PCA) media. Among these media, 1 M NaCl and 1 M sorbitol provide hyperosmotic stress conditions for fungal growth; 0.1% SDS is used to mimic the cytoplasmic membrane (Zheng et al., 2012); and V8 and PCA provide rich and poor nutrition for fungal growth, respectively. Compared with CSG2-3-F10, CSG2-3 showed a clearly reduced growth rate in 1 M sorbitol, 0.1% SDS and V8 media, except in the other two media (**Figures 5F, G** **)**. This result indicated that the presence of the virus is detrimental to the growth of host fungi and may also regulate the integrity of the cell wall. In total, these results suggested that dsRNA increases appressorium formation but reduces conidia production.

### Statistics on the numbers of differentially expressed genes

**Figure 6:**
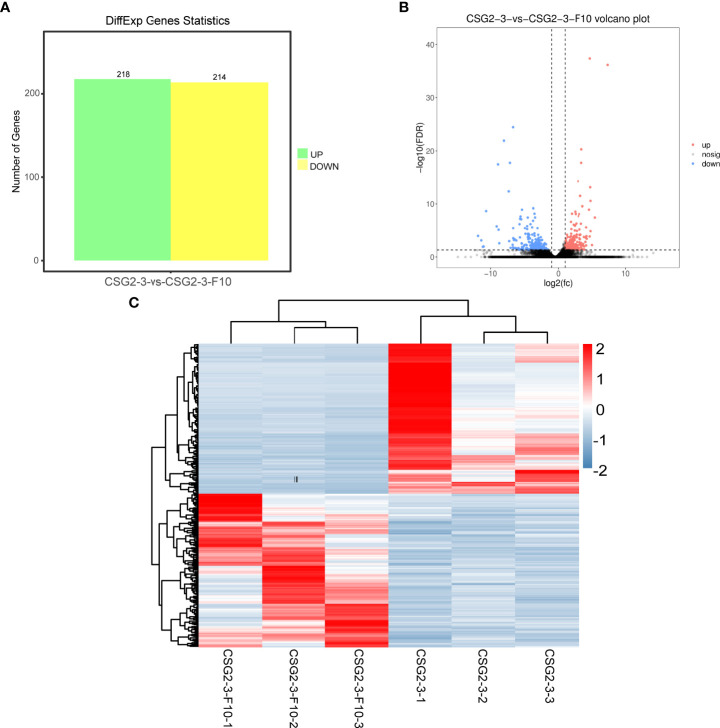
Differentially expressed genes between CSG2-3 and CSG2-3-F10. **(A)** Number of DEGs between CSG2-3 and CSG2-3-F10. The green column represents upregulated DEGs, and the yellow column represents downregulated DEGs. **(B)** The volcano plot of DEGs between CSG2-3 and CSG2-3-F10 displays –log10 (FDR) on the Y-axis, index of gene differences from NOIseq, and Log2 (fold-change (FC)-value) on the X-axis. The pink dots represent upregulated DEGs, and blue dots represent downregulated DEGs between CSG2-3 and CSG2-3-F10. Black dots indicate genes without statistical significance. **(C)** Heatmap of DEGs between CSG2-3 and CSG2-3-F10. The abscissa represents the CSG2-3 vs. CSG2-3-F10 differential genes, and the left lines represent the clustering relationship between genes. The above lines represent the clustering relationship between samples. After Z-SCOR homogenization of gene expression, the color changed from blue to red, indicating that the expression of this gene in this sample changed from low to high.

To identify the number of differentially expressed genes (DEGs) regulated by viruses between the virus-infected *C. fructicola* strain CSG2-3 and the virus-free strain CSG2-3-F10, transcriptome sequencing of these two strains was carried out in this study. Data analysis consists of three parts as described by (Love et al., 2014). First, read counts were normalized; then, hypothesis testing probability (*P* value) was calculated according to the model; finally, multiple hypothesis testing and correction were performed to obtain the false discovery rate (FDR) value. DEGs were screened out between the treatments with log_2_ FC (fold change) > 1 and FDR< 0.05. A total of 432 DEGs were identified between CSG2-3 and CSG2-3-F10, including 218 upregulated genes and 214 downregulated genes (**Figure 6A**). The volcano plot and heatmap show the distribution of DEGs and the relative expression levels of DEGs, respectively (**Figures 6B, C**).

### GO enrichment analysis of DEGs

**Figure 7:**
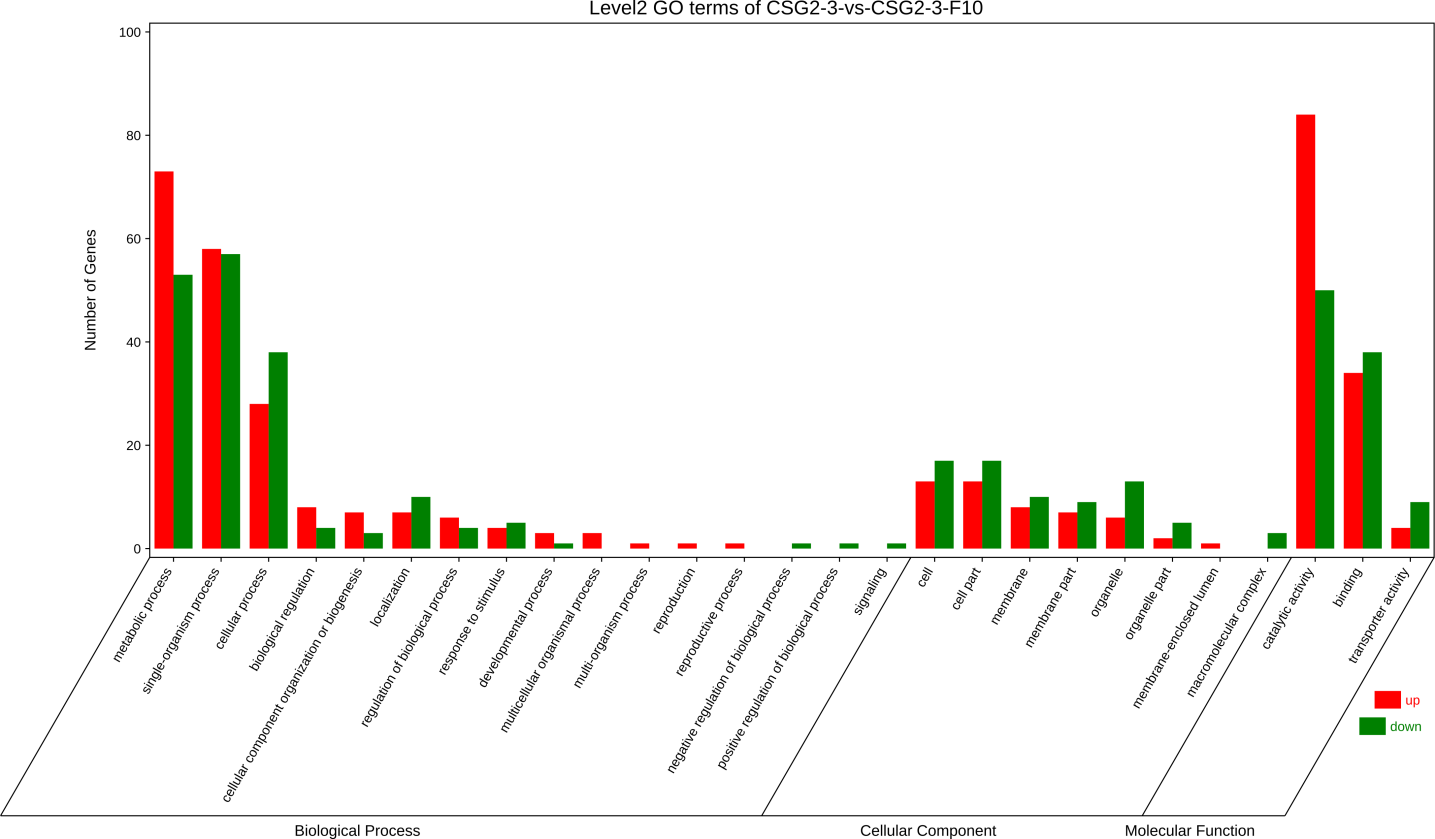
Gene Ontology (GO) enrichment analysis of DEGs between CSG2-3 and CSG2-3-F10.

Furthermore, Gene Ontology (GO) enrichment analysis of DEGs between CSG2-3 and CSG2-3-F10 was conducted. According to the analysis, genes were divided into three categories: biological process, cellular component, and molecular function. Among these categories, biological process was the most enriched category. Meanwhile, in biological processes, most functional classifications of DEGs were located in metabolic processes, single-organism processes and cellular processes. However, in the cellular component category, the functional classification of DEGs was more average, such as cell, cell part, membrane, membrane part, and organelle. However, in the molecular function category, the functional classification of DEGs was located mainly in binding and catalytic activity (**Figure 7**). These functional enrichment results indicated that these functions are essential for the life activities of *C. fructicola* and that the virus might be involved in regulating these processes, such as by regulating the internal components of the cell membrane to promote the invasion of the virus (Gao et al., 2020).

### KEGG pathway enrichment analysisof DEGs

**Figure 8:**
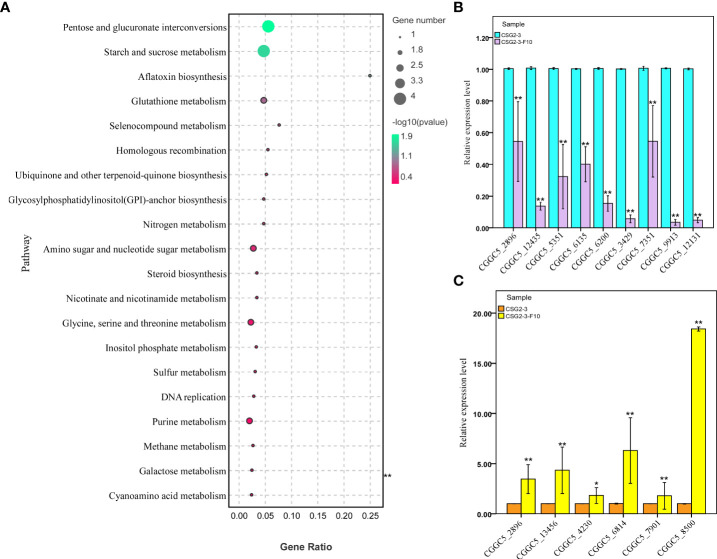
KEGG enrichment analysis and RT-qPCR verification of DEGs between CSG2-3 and CSG2-3-F10. **(A)** The bubble graph displays KEGG items on the Y-axis and rich factors on the X-axis. The color of bubbles represents the P value. The size of bubbles represents the number of genes. **(B, C)** The expression of fifteen DEGs (nine downregulated and six upregulated DEGs) was examined *via* RT-qPCR. Biological and technical replicates were performed for each RT-qPCR. The asterisk represents a significant difference (Student’s *t*-test, ‘**’, P<0.01, ‘*’, P<0.05).

In organisms, the Kyoto Encyclopedia of Genes and Genomes (KEGG) pathway-based analysis is helpful to further understand the biological functions of genes. KEGG is the main public database on Pathway (Kanehisa et al., 2016). In our study, KEGG enrichment analysis was used to determine the specific pathways involved in DEGs between the virus-infected *C. fructicola* strain CSG2-3 and the virus-free strain CSG2-3-F10. As a result, among these DEGs, only 34 DEGs were involved in 41 KEGG pathways, which is much lower than we expected. In addition to these KEGG pathways, pentose and glucuronate interconversions (ko00040) and starch and sucrose metabolism (ko00500) were the two most enriched pathways, followed by glutathione metabolism (ko00480) (**Figure 8A**).

Genes related to carbohydrate metabolism are downregulated: carbohydrates play an important role in the pathogenesis of fungi. In the KEGG pathway analysis, the two most enriched pathways with a total of 8 DEGs were related to carbohydrate metabolism, including CGGC5_11093, CGGC5_14142, CGGC5_6059, CGGC5_5532, CGGC5_5757, CGGC5_5992, CGGC5_10314 and CGGC5_6991. Compared with CSG2-3-F10, the expression levels of these related genes in CSG2-3 were significantly downregulated, except for CGGC5_6991. We speculated that these results may be consistent with the attenuated pathogenicity characteristic of CSG2-3.

Moreover, we also found several interesting pathways, such as aflatoxin biosynthesis (one gene), homologous recombination (one gene), DNA replication (one gene), and RNA degradation (one gene). The function of these genes has yet to be found.

In addition, we conducted statistical analysis on amino acid metabolism-related genes, focusing mainly on tyrosine metabolism-related genes and reduced glutathione metabolism-related genes. Three genes related to amino acid metabolism were found, including CGGC5_4624, CGGC5_3253 and CGGC5_13673. The expression of the former gene CGGC5_4624 was upregulated in CSG2-3, while the latter two genes were downregulated.

### Verification of transcriptomic data by quantitative PCR

To verify the reliability of the transcriptome data, we randomly selected 15 DEGs for RT-qPCR, including formate dehydrogenase (CGGC5_3890), C6 transcription factor (CGGC5_4230, CGGC5_7351, CGGC5_7901), integral membrane protein (CGGC5_6814), membrane-associated proteins in eicosanoid and glutathione metabolism (CGGC5_8510), RAM signaling pathway protein (CGGC5_13456), adenosine triphosphate (ATP)-dependent RNA (CGGC5_2896), zinc finger protein (CGGC5_3429, CGGC5_5351), alpha-mannosyltransferase (CGGC5_6135), protein kinase domain protein (CGGC5_6200), protein kinase domain-containing protein (CGGC5_9913, CGGC5_12131), and S-adenosine methionine (SAM)-dependent methyltransferase (CGGC5_12435). The results of RT-qPCR were consistent with the transcriptome data and confirmed the reliability of the transcriptome data (**Figures 8B, C** **)**.

## Discussion

In this study, two viruses, designated CfOLV1-CgOLV1 and CfOLV2, were identified in the plant pathogenic fungus *C. fructicola*. Moreover, virus elimination and horizontal transmission confirmed that hypovirulence of CSG2-3 was due to dsRNA infection, and we also found that the dsRNA affected appressorium formation of its host *C. fructicola*, which inhibited pathogenicity of *C. fructicola* (O'Connell et al., 2012).

Coinfection of a single fungal host by multiple varieties of viruses is a common phenomenon; hence, there are various types of virus-virus interactions in fungi, including synergistic interactions, mutualistic interactions and antagonistic interactions (Hillman et al., 2018). For example, Wu et al. (2017) found that a hypovirulence-associated mycovirus, SsMYRV4, inhibits host nonself recognition by regulating the expression of reactive oxygen species (ROS)-related genes, thus facilitating the horizontal transmission of the heterologous virus (Wu et al., 2017). A +ssRNA virus (YkV1) could hijack the capsid protein of the coinfected dsRNA virus YnV1 and replicates as a dsRNA virus in the phytopathogenic fungus *Rosellinia necatrix* (Zhang et al., 2016). Another coinfection example is that only the viruses RnMBV2 and RnPV1 infecting the host fungus *Rosellinia necatrix* simultaneously could lead to hypovirulence (Sasaki et al., 2016). In the process of dsRNA extraction, we found an additional segment, dsRNA-M, in addition to CfOLV1-CgOLV1 and CfOLV2. Strikingly, neither conventional nor high-throughput sequencing helped us to acquire further viral genome information. Therefore, we hypothesized that dsRNA-M could be a satellite RNA or satellite-like RNA of CfOLV1-CgOLV1 or CfOLV2. Previous studies proved that satellite RNAs or satellite-like RNAs usually rely on their helper virus for both replication and encapsulation and inhibit helper virus genome replication for their own replication. Therefore, satellite RNAs and satellite-like RNAs have the ability to alter the disease symptoms caused by helper viruses (Roossinck et al., 1992; Shimura et al., 2011; Xu et al., 2016). Since the sequence information of dsRNA-M is incomplete, we attribute the reason to the possible absence of a segment of dsRNA-M in CSG2-3-Y7-D3 and CSG2-3-Y7-D4. Further research is needed to confirm this hypothesis. For example, protoplast elimination and infection cDNA clone are both feasible methods for subsequent research. In addition, it is also meaningful to investigate whether there is any interaction among CfOLV1-CgOLV1, CfOLV2 and dsRNA-M, which will provide a new perspective on the diversity of virus-related RNAs in *C. fructicola*.

Similar to most pathogenic fungi, conidia and appressorium play a key role during *C. fructicola* infection (Perfect et al., 1999). However, in our research, although the virus-infected CSG2-3 displayed a much greater proportion of appressorium formation than CSG2-3-F10, CSG2-3 clearly showed hypovirulence. This phenomenon appears contradictory; one of the reasons may be that the concave slides do not provide the correct signaling molecules, such as cutin and wax (Li et al., 2020). There is a similar case: the *M.oryzae* zinc finger protein mutant *cso1* is markedly deficient in melanin pigmentation but can cause visible symptoms on unwounded leaf blades and sheaths of rice (Zhou et al., 2009). During the infection process, *Colletotrichum* fungus can form melanized appressoria that directly penetrate host epidermal cells by producing a penetration peg. In *Colletotrichum lagenarium*, the melanization of appressoria is crucial for appressorial function, and three melanin biosynthesis enzyme genes, one regulatory gene, have been characterized (Kubo and Furusawa, 1991; Takano et al., 1995; Kubo et al., 1996; Perpetua et al., 1996; Tsuji et al., 2000; Tsuji et al., 2003). However, *LAC2*, a gene encoding a secreted laccase, was found to be involved in appressorial melanization and pathogenicity (Lin et al., 2012). These studies indicated that melanization of appressoria is a very important pathogenicity factor of *Colletotrichum*. In fact, we found that melanization of appressoria was obviously observed in CSG2-3 but not CSG2-3-F10 (**Figure 5A**). This led us to wonder whether the melanization of appressorial can actually infect the fungal pathogenicity between CSG2-3 and CSG2-3-F10. Combining the above research, conidia, melanin and appressoria do not seem to be determinative factors of pathogenicity.

In transcriptome data, we found that eight DEGs associated with carbohydrate metabolism were significantly downregulated in CSG2-3. These carbohydrate metabolisms may be essential for fungal life activities; therefore, the downregulation of these related genes resulted in obvious hypovirulence, which was consistent with the CSG2-3 inoculation results. However, previous studies have proven that melanin is associated with resistance to adverse living conditions and the infection capacity of fungal hosts (Cao et al., 1992; Fan et al., 2017). In this study, the appressorium of CSG2-3 showed marked melanin deepening, but the pathogenicity was much lower than the pathogenicity of CSG2-3-F10. We assume that the reason for this phenomenon is caused by the high expression of the tyrosine metabolism-related gene CGGC5_4624 in CSG2-3. Interestingly, melanin does not appear to be strongly associated with the pathogenicity of pathogenic fungi, which causes both positive and negative regulation in different fungi (Steiner and Oerke, 2007; Zhang et al., 2015; Li et al., 2019b).

In conclusion, in order to accurately character the causes of hypovirulence in CSG2-3, multiple approaches are needed simultaneously in future. Such as protoplast elimination, infection cDNA clones, and direct sequencing of dsRNA-M after gel cut elution. The manifestation of pathogenicity in fungal infection is the final form, with appressorium and melanin as intermediates. Whether the two most enriched pathways (pentose and glucuronate interconversions, starch and sucrose metabolism) are participated in the regulation of fungal pathogenicity and whether mycoviruses control fungal pathogenicity by regulating infection-related genes, involved multiple aspects and factor of multiple regulatory networks that deserve to be investigated.

## Data availability statement

The datasets presented in this study can be found in online repositories. The names of the repository/repositories and accession number(s) can be found below: https://www.ncbi.nlm.nih.gov/, MW300427 and MW300428.

## Author contributions

JG and XZ contributed equally to this work. All authors contributed to the article and approved the submitted version.

## Funding

This research was supported by the Hunan Provincial Natural Science Foundation of China (2021JJ30356), Hunan Provincial Key Research and Development Program of China (2020NK2045), and Hunan Provincial Science and Technology Innovation Platform Construction Fund (20K071).

## Acknowledgments

The CSG2-3 strain in the manuscript was generously provided by Dr. Zhengbing Zhang (Plant Protection and Inspection Station, Hunan Agricultural Department, Changsha City, Hunan Province, 410005, P.R. China). We thank all the authors for their support of this article.

## Conflict of interest

The authors declare that the research was conducted in the absence of any commercial or financial relationships that could be construed as a potential conflict of interest.

## Publisher’s note

All claims expressed in this article are solely those of the authors and do not necessarily represent those of their affiliated organizations, or those of the publisher, the editors and the reviewers. Any product that may be evaluated in this article, or claim that may be made by its manufacturer, is not guaranteed or endorsed by the publisher.
