# Supplementary material for: Hypovirulence caused by mycovirus in Colletotrichum fructicola
Source: Front Plant Sci. 2022 Oct 7;13:1038781. doi: 10.3389/fpls.2022.1038781 (PMC9585321; doi:10.3389/fpls.2022.1038781)
Supplement: Supplementary file 1 [file Table_1.docx]

Sequences of Colletotrichum fructicola ourmia-like virus 1- Colletotrichum gloeosprioides ourmia-like virus 1 and Colletotrichum fructicola ourmia-like virus 2 (CfOLV2)

> Colletotrichum fructicola ourmia-like virus 1- Colletotrichum gloeosprioides ourmia-like virus 1 (CfOLV1CgOLV1)

TTGGATGTCAAAAAATCCTTGACTCCCCCACACTCCACGAGTATGGGTCAAAAAAGGTCTGATCATGTAAAAACGCCTGCGTTTTGGTCAGCTACCTGTTCGGTTCGCCGTCGATTTCTACGCTTGAAGCGGGCTCTCGAGAAACTTCATCAAGTTTCTCTTCCCTCTCCAATGTTGGTAGGAACGGCAGAATCGAAAGAAAAGAACTTCAAAAGATTTCTTAGTGATCTTATTGAAGGTAAAGAACACATTTGGGTGAATCCACTAAGGAGACTACAGCGCAGAACACGCTATAGTATCGGACTCACCCTCTTTCTCTTTAGGAAAATTCTTAAGGGTAAGTCACCTGACTTAGAGTCACTTCTGGAACGAGTCTCATCTCCCCCCGGGGAGGTGGATAATGACTTCCTTGAGTTTGCTCGTGATCAGGTGGATCGTATCTTCCCACAGGGATGGGACCGCGGTTACTGGAACCGTGTCAAATCCGCGACTGTGAGTACGAAGTCTTGCTCTGAATCTAGAAGATCGGATGGAGGTAATAGAATGTATTGGTTAGACAGAGCGAACTCGGATGCCAGGTCCGAGTTCTGTCAATACCTGTCAGGTGAACGCGATCTTCGGGATCTGCCTGACACTGCTGCCCAATTCATCAACGTGGAGACTTCAGGTAAGGTCCGCGCCTTATCTGTGCCTCCGGCAAAGTTTTCACTTCTATTACCCTTACATAAATTGATGTATGATCAACTCAGTAAAGAACCCTGGCTTCTCCGAGGAGATGCCAGTGCGAAGAGTTTCAGCGACTTCCTAAAGCAGGAAGGTGAACTATTCGTTAGTGGTGATTATGAGTCGGCAACAGACAATTTGAATCCCATCATTCAACACGAAATTCTCTCTAAACTTCTCAGCAACTGTACCGAGGTCCCCTCGGGGATCTCGCAGCTTGCTTTGCGGTCTCTTCATTGGCGAGCCGGTGTTCGCGGGAACATCGACGCTTCTGATGAAGAGCATCCTCCGGATATTAGAGAGATTACCAGTGGACAGATGATGGGTTTCCCGATCTCCTTTCCCCTCCTGTGTCTTATTAATTATATTACTTTTAAGTTTGCCGTTAGGCGACACGTCCCACTGAGAATAAATGGGGACGATATTGTTGCTAGGATAACGAAAGAAGAATATGATCGATGGGTCACCCTGGTGGGTAAGAGTGGTCTTAAGTTGTCGGTTGGTAAGACTATGGTGGACAGAAGTTTCTTCACACTGAACTCGTCCCTCTTCGAGGGGAAGAAGGTCCGGGTCGTGGAGTTACCTTTTATCCGCTCGAAGGCTTTTTTTGGTCTCACCGAAGAAGATAAGGAAGGTTCACCTTATTCTTTTGTAGGCAGGTACAAGTCCTTCTGTCCCGGGTACTTTGGACAGAAGAGGTGGTACCTAAGGACCTTGTTCCTCCAGGGGAATAGGGATCAGATCATGAAAAGTGGTGGAAGTCTCAACCGACGATTCCAAATGAATATTCCTGCTTGGGTCCTTCGGGACTCCCAGCTGTTGTCGAGAGAAGCCTCGTTTCTTGATTTACCAGCTGAGAAGCCCCCTCCAATGGAGAAGAGCGAATGGTCGAAACGCCCGAAGGGCTTCAGAATCGCTTATTCTGCTTCTAAGAAGGAATATTCGGAGGAAGAGAAAGAGGAGTTGATTGAGGCTCAGGTCCAATCAGCTTGGGAAGGTCCCGACGAGGACGAAGACTATCGTCAGGTTTTCGAAGGCCTGAAAGATGTTTCTTTTAATCTGATCAAGATGTCTAAGATGTTGAGGCTCCCCGTCGGGGAGCTGAGGAAGACATTGAGGGCTAGAGCAGAGAAGATAGTTTCATCTTACAAGAGACCGGCGAAAGTCTATCCCTACTGGAAGAAAAGTGAAGGTAGCTGTCATGCTTCACTCGCATGTCAGGAAGAACAAGATCAGCAGGTGGTGGAGCCCAGGCTCTACCCCCCACCATTAGTCTACTGATAGGAGGGCCAGAAGAGATAGGTATGTTGAGCCTGACGGCTGGATAAACCGTGCCATTCGGTTGGACCGGAACCTACCCTGAATCAACAGGGTGCCTTGCATACTGATTCTCCAGCCGGGGGTGTTGGACTTAACCACGAACCAACCGGTAGAATCTCAACCCCGCTGTAACAGGCGGTCCAGTAGAGATTCATCTCGAGAGAGATAATGCAGTTCCTCTGCAGGATTACGGAGAGGGGGGTAACGAATGGCGTTGTGCAACATGTCGCACACGTTGTCCCTGTCACTAAGTATAGCATCCCTTCGGGCGATGATCTCAGTAAACAACTGGGCTAAGTAGTCCTAGAAGTAGAGAATGGATGGGAGCCTGCTCCCGTGGCTAGCGCGCGCGACACCAACCAGGGCGTCTAATTAAATACTGGAGGCGATGGTTCGCTGAGGGGGAAGCGGTCCCAACGGTTCGATTCCGAAGGGTCTCCCGGATATTGTCTACGGATGGTATCAAACCACCA

> Colletotrichum fructicola ourmia-like virus 2 (CfOLV2)

AGCTACAGGAGATAAATCCCGTTAGATAAGCGCAGTAGCCTGACTTATCTATCATGTTAGCCTCACGGCCAACGAAACGTAGCTCCCGTCCCCCAACCAGTAGGTTAGGTGGGAACTCCCGAGTTAATTCCGCTAGCGGTTCTCGGGCTAAGCGGTGTGTTGACTGCTCTCGGGCAGAACGTCACACCAAGGAAACGGTCCACAATGGTATGCTTCTCATTCGGCTCAGGTTCGGTCTTCCTAACTCTGAGTTACCGGATTGCAATCCATCTGAACTAGGACGTTTCCTTTCTTTTCTTTTGCTACAGGGCAAGGAGCGGGCCTCTGTAGCTTTCCCTAGACGCCAGGTCAATAGGGAAGACGGTCTTTGTAACTTGCAAAGATTGTGCCGTCGAGATAGGTGGGAACTGGCCCATGCCTTATCGTCAATAAAACGTAACCTGCCGGCAGGTTGCGTACAGCACACTCCGTCGTCGCGTTTATTGTGGGAGGAAAACGCGACCTCAAAACCTCCCCCCTCATCCTCTGAGTTCCTAGCCTTCGTCAAGAAGGAGATCACTCGGATCTTTCCGCCAGGGTGGGATCGGAATTATGGCTCCTTTGTCGGGAGCCACTTGCCCAATCCTACCTCCCGGTATAATAAGGATAAGCCTGCTGACTTGGCATGGCTTAACCGGCGGTCTGATTTCTTTACCTGCGCCCTCCAGGAGACAGAAGTGTCACCTGTCTTTTGTGCGAGGTACAAAGAAGTCCTTTCTGCAGGCAAGTGTAGGCCTCTCCTCATATACGATGAGAAGATTGATCTGCTTGCCCCTCTTCACAAACTGATATACTCTCATATCAGGAAGACTGATTGGCTTCTGTGCGGTCCACCGACCGAAAAAAGGATGAAATCTGTCTGTGTCAAAGCCTACCAAACCTCAGTTGATTTGGTAGCGGCGACAGACAACCTCCGACATGATGTCGCGGATGAGATCCTCGATGCCATGTTCTTCACCTCAGTGAAGATTCCGAGATCCGTGAGAGCTTTGGCTCATGGGTCGCTGAGCCCTTTGTTTAAGGACTCTAGGAATATATGGAGGCGCGTCCGTCACGGACAGATGATGGGGGCCTACCTCTCCTTTCCTCTGCTTTGTCTTCAGTCATACCTAGCGGCCAGGTGGGCTACCAGGTTGGAAGAGGGAGCTCGTATCCTGATTAATGGGGATGACTGTGTCATCTCAGCTGATCGGATCATCACTAAAGAAGATTACCCGCGAGGGTTTATTCTTAATGATAATAAGACAATACGAGCTCCTAATGTTGTAGAAATTAATTCTACAGCATTCATAAGGCAGGGGAAAAGGTGGCGCGAAGTACGCCATCTCAGGAGAGGAGGAGCGGTGTCCGATTATGTCGGCGTGAGGCACATGGCTGAGGCGGTTAGGGATACTCCCTACGAACCTGCCTTCCAACGTGCCCGGATCGGACGCCACTGGGGTTTCATGCCCTCTCAACTAGGACATAGGTCCTATCCAGCGTTTTTGAGAGAACGCGGAATGGTAAAACGGGTTTTTACTCCTCTCCCCGAGAGGAAAGTGAGCCGTGATACCAGGTTGAGAATTTATAGGGGAGAGCCTAGCGAGGTTGAGAAGGAAGTTCTTCGGAGCTTCCTCTGGACCTACGGGCGTGTCCCCGGGCAGAAGAGAGACGTATTTAACCCAACCCACGGTTATGTACGTCGGACATACGGATATAGGTCCCGGCCCTATATCTACAGACTCAGCTATGTCAACTGGGCCTGTAAGCTGGACCCTCCAGCTCGTAAAAAGGACCAGGCGTATTTCCTACCAGAGGAATTTATACCTGAAGAAGAACAGAAAGGTCTGAATGATCTCGCTACGTTCGGAGATCACTGGTATCCAGAGGACTAGTTCAGTCCAAACCGTTTCAGGCCGGTTGTGTTCGGCAGACATAATGTTAGCCGTTCGTCGCGGGGCTCTCAGAGCAGGACAACCGGGCCACGCCCTTACCATCACCGGGATGGAAGGAAATTTAGCGTCGTGGCT
